# Bis(μ_3_-pyrimidine-4-carboxyl­ato)bis­(μ_2_-pyrimidine-4-carboxyl­ato)tetra­kis­(aqua­lithium)

**DOI:** 10.1107/S160053681203142X

**Published:** 2012-07-14

**Authors:** Wojciech Starosta, Janusz Leciejewicz

**Affiliations:** a Institute of Nuclear Chemistry and Technology, ul. Dorodna 16, 03-195 Warszawa, Poland

## Abstract

The asymmetric unit of the title compound, [Li_4_(C_5_H_3_N_2_O_2_)_4_(H_2_O)_4_], contains two symmetry-independent Li^I^ ions, two symmetry-independent ligands and two symmetry-independent coordinated water mol­ecules. They form a dinuclear unit in which the two Li^I^ ions are bridged by two carboxyl­ate O atoms from the two ligands. Two dinuclear units related by an inversion centre form the tetra­meric mol­ecule. One of the Li^I^ ions shows a distorted tetra­hedral coordination geometry, the other a distorted trigonal–bipyramidal environment. The tetra­mers are held together by hydrogen bonds in which coordinated water mol­ecules act as donors, and the carboxyl­ate O atoms act as acceptors. A hydrogen bond between coordinated water molecule as donor and a ring N atom as acceptor is also observed.

## Related literature

For the crystal structures of four 3*d* metal complexes with pyrimidine-4-carboxyl­ate and aqua ligands, see: Aakeröy *et al.* (2006 ▶). For the structure of an ionic Li^I^ complex with pyridazine-3,6-dicarboxyl­ate and water ligands, see: Starosta & Leciejewicz (2012 ▶).
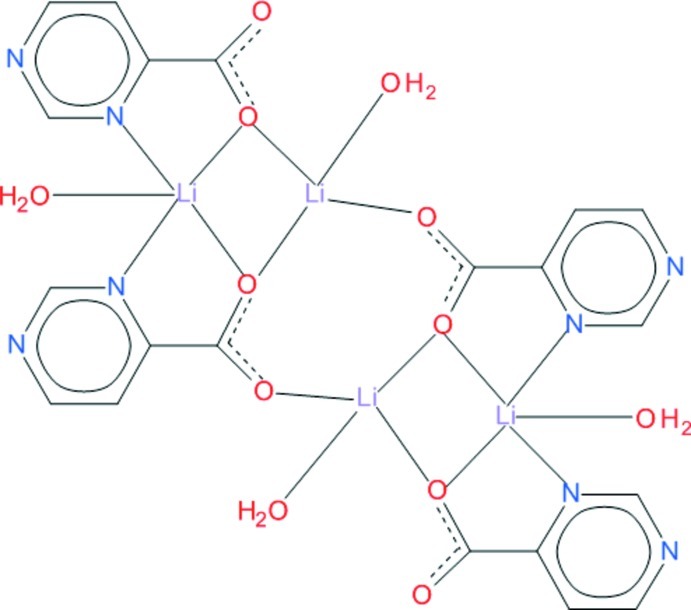


## Experimental

### 

#### Crystal data

[Li_4_(C_5_H_3_N_2_O_2_)_4_(H_2_O)_4_]
*M* *_r_* = 592.20
Triclinic, 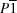

*a* = 7.2750 (15) Å
*b* = 7.9108 (16) Å
*c* = 12.966 (3) Å
α = 77.91 (3)°
β = 84.59 (3)°
γ = 67.23 (3)°
*V* = 672.7 (2) Å^3^
*Z* = 1
Mo *K*α radiation
μ = 0.12 mm^−1^
*T* = 293 K
0.24 × 0.20 × 0.08 mm

#### Data collection

Kuma KM-4 four-cricle diffractometer
Absorption correction: analytical (*CrysAlis RED*; Oxford Diffraction, 2008 ▶) *T*_min_ = 0.982, *T*_max_ = 0.991
4167 measured reflections
3874 independent reflections
2417 reflections with *I* > 2σ(*I*)
*R*_int_ = 0.048
3 standard reflections every 200 reflections intensity decay: 2.8%

#### Refinement

*R*[*F* ^2^ > 2σ(*F* ^2^)] = 0.044
*wR*(*F* ^2^) = 0.164
*S* = 1.07
3874 reflections
216 parameters
H atoms treated by a mixture of independent and constrained refinement
Δρ_max_ = 0.50 e Å^−3^
Δρ_min_ = −0.43 e Å^−3^

### 

Data collection: *KM-4 Software* (Kuma, 1996 ▶); cell refinement: *KM-4 Software*; data reduction: *DATAPROC* (Kuma, 2001 ▶); program(s) used to solve structure: *SHELXS97* (Sheldrick, 2008 ▶); program(s) used to refine structure: *SHELXL97* (Sheldrick, 2008 ▶); molecular graphics: *SHELXTL* (Sheldrick, 2008 ▶); software used to prepare material for publication: *SHELXTL*.

## Supplementary Material

Crystal structure: contains datablock(s) I, global. DOI: 10.1107/S160053681203142X/kp2430sup1.cif

Structure factors: contains datablock(s) I. DOI: 10.1107/S160053681203142X/kp2430Isup2.hkl

Additional supplementary materials:  crystallographic information; 3D view; checkCIF report

## Figures and Tables

**Table 1:** Selected bond lengths (Å)

Li1—O11	1.961 (3)
Li1—O22	1.932 (3)
Li1—O21^i^	1.953 (3)
Li1—O1	1.967 (3)
Li2—O11	2.021 (3)
Li2—O22	2.020 (3)
Li2—N13	2.155 (3)
Li2—O2	1.998 (4)
Li2—N23	2.205 (3)

Symmetry code: (i) 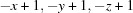.

**Table 2:** Hydrogen-bond geometry (Å, °)

*D*—H⋯*A*	*D*—H	H⋯*A*	*D*⋯*A*	*D*—H⋯*A*
O1—H1⋯O12^ii^	0.83 (3)	1.98 (3)	2.8120 (18)	175 (2)
O2—H3⋯O12^iii^	0.93 (3)	1.84 (3)	2.7671 (18)	177 (3)
O1—H2⋯O21^iv^	0.87 (3)	1.98 (3)	2.7941 (18)	155 (3)
O2—H4⋯N21^v^	0.81 (4)	2.10 (4)	2.8881 (19)	166 (3)

Symmetry codes: (ii) 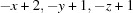; (iii) 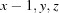; (iv) 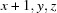; (v) 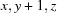.

**Fig. 1.**
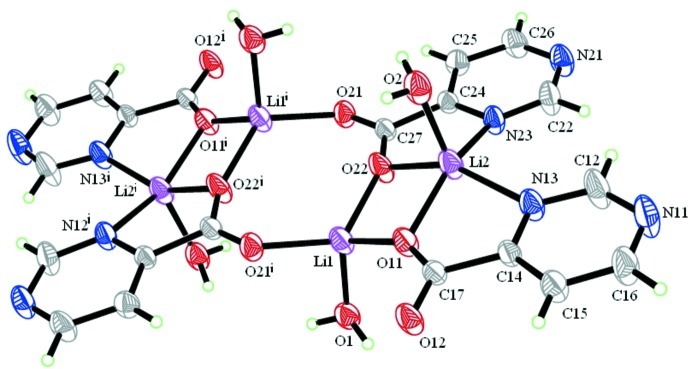
The tetrameric structural unit of the title compound with atom labelling scheme and 50% probability displacement ellipsoids. Symmetry code: (i) -x + 1, -y + 1, -z + 1.

**Fig. 2.**
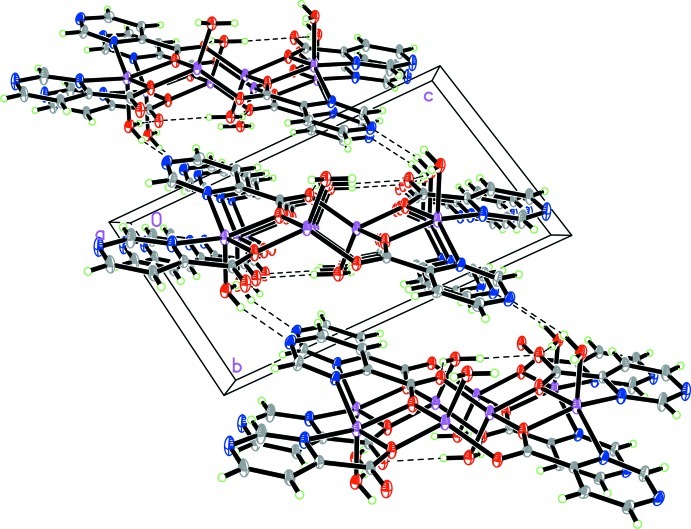
Packing of the tetramers with hydrogen bonds marked by dashed lines.

## supplementary crystallographic information

### Comment

The structure of the title compound is built of tetrameric molecular clusters, each composed of two dinuclear units related by an inversion centre. A dinuclear unit consists of two symmetry independent Li^I^ ions, two symmetry independent ligand molecules and two symmetry independent coordinating water molecules (Fig. 1). The ligand molecule PMC1 shows µ_2_ coordination mode: its carboxylato O11 atom acts as bidentate bridging to Li1 and the Li2 ions, leaving the O12 atom chelating inactive. Ligand PMC2 shows µ_3_ bridging mode since both its carboxylato O atoms act as bridging: the O22 atom bridges the Li2 ion and the Li1 ion while the O21 atom links the Li2 ion and the Li1^(i)^. The Li1—O11—Li2—O22—Li1 bridging pathway forms a core of a dinuclear unit with r.m.s. of 0.0133 (2) Å which through bridging O21 and O21^(i)^ atoms generates a centrosymmetric tetrameric molecular cluster with a core represented by a bonding loop Li1—O22—C27—O21—Li1^(i)^—O22^(i)^—C37^(i)^—O21^(i)^—Li1^(i)^ with r.m.s. of 0.1664 (3) Å. Li1 ion, chelated by O1, O11, O22 and O21^(i)^ atoms shows distorted tetrahedral coordination geometry. On the other hand, Li2 coordination polyhedron is a distorted trigonal bipyramid with an equatorial plane composed of O11, O2 and N23 atoms. The Li2 ion is 0.1809 (2) Å out of this plane; N13 and O22 atoms are at axial positions. The observed Li—O and Li—N bond distances are typical (Table 1). Both pyrimidine rings are planar with r.m.s. of 0.0080 (2) Å and 0.0079 (2) Å for ligand PMC1 and PMC2, respectively. The carboxylate groups C17/O11/O12 and C27/O21/O22 make dihedral angles of 4.3 (1)° and 3.3 (1)/% with the respective rings. Bond distances and bond angles within both ligand molecules do not differ from those observed in the structures of 3 d-metal complexes with the title ligand (Aakeröy *et al.*, 2006). A hydrogen bond system in which water molecules are donors and carboxylate O atoms are acceptors is responsible for the cohesion of the structure (Table 2, Fig. 2). Isolated neutral tetrameric clusters with a different internal structure have been detected in the structure of an ionic Li^I^ complex with pyridazine-3,6-dicarboxylate and water ligands aside dimeric anions and hydrazine cations (Starosta & Leciejewicz, 2012).

### Experimental

1 Mmol of pyrimidine-4-carboxylic acid dissolved in 20 ml of water was titrated with an aqueous solution of LiOH until pH of 5.6 was reached. Then the mixture was boiled under reflux with stirring for 5 h. Left to crystallize at room temperature, well shaped single-crystal plates were found after evaporation to dryness. They were washed with cold metanol and dried in air.

### Refinement

Water H atoms were located in a difference map and refined isotropically, while the H atoms attached to pyrimidine C atoms were located at a calculated position and treated as riding on the parent atom with C—H=0.93 Å and *U*_iso_(H)=1.2*U*_eq_(C). Residual electron density values can be explained as due to the decomposition of the single-crystal sample (observed decay 2.8%).

### Crystal data

**Table:**

[Li_4_(C_5_H_3_N_2_O_2_)_4_(H_2_O)_4_]	*Z* = 1
*M**_r_* = 592.20	*F*(000) = 304
Triclinic, *P*1	*D*_x_ = 1.462 Mg m^−^^3^
Hall symbol: -P 1	Mo *K*α radiation, λ = 0.71073 Å
*a* = 7.2750 (15) Å	Cell parameters from 25 reflections
*b* = 7.9108 (16) Å	θ = 6–15°
*c* = 12.966 (3) Å	µ = 0.12 mm^−^^1^
α = 77.91 (3)°	*T* = 293 K
β = 84.59 (3)°	Plate, colourless
γ = 67.23 (3)°	0.24 × 0.20 × 0.08 mm
*V* = 672.7 (2) Å^3^	

### Data collection

**Table:**

Kuma KM-4 four-cricle diffractometer	2417 reflections with *I* > 2σ(*I*)
Radiation source: fine-focus sealed tube	*R*_int_ = 0.048
Graphite monochromator	θ_max_ = 30.1°, θ_min_ = 1.6°
profile data from ω/2θ scans	*h* = 0→9
Absorption correction: analytical (*CrysAlis RED*; Oxford Diffraction, 2008)	*k* = −10→10
*T*_min_ = 0.982, *T*_max_ = 0.991	*l* = −18→18
4167 measured reflections	3 standard reflections every 200 reflections
3874 independent reflections	intensity decay: 2.8%

### Refinement

**Table:**

Refinement on *F*^2^	Secondary atom site location: difference Fourier map
Least-squares matrix: full	Hydrogen site location: inferred from neighbouring sites
*R*[*F*^2^ > 2σ(*F*^2^)] = 0.044	H atoms treated by a mixture of independent and constrained refinement
*wR*(*F*^2^) = 0.164	*w* = 1/[σ^2^(*F*_o_^2^) + (0.1119*P*)^2^] where *P* = (*F*_o_^2^ + 2*F*_c_^2^)/3
*S* = 1.07	(Δ/σ)_max_ < 0.001
3874 reflections	Δρ_max_ = 0.50 e Å^−^^3^
216 parameters	Δρ_min_ = −0.43 e Å^−^^3^
0 restraints	Extinction correction: *SHELXL97* (Sheldrick, 2008), Fc^*^=kFc[1+0.001xFc^2^λ^3^/sin(2θ)]^-1/4^
Primary atom site location: structure-invariant direct methods	Extinction coefficient: 0.045 (10)

### Special details

**Table:**

Geometry. All e.s.d.'s (except the e.s.d. in the dihedral angle between two l.s. planes) are estimated using the full covariance matrix. The cell e.s.d.'s are taken into account individually in the estimation of e.s.d.'s in distances, angles and torsion angles; correlations between e.s.d.'s in cell parameters are only used when they are defined by crystal symmetry. An approximate (isotropic) treatment of cell e.s.d.'s is used for estimating e.s.d.'s involving l.s. planes.
Refinement. Refinement of *F*^2^ against ALL reflections. The weighted *R*-factor *wR* and goodness of fit *S* are based on *F*^2^, conventional *R*-factors *R* are based on *F*, with *F* set to zero for negative *F*^2^. The threshold expression of *F*^2^ > σ(*F*^2^) is used only for calculating *R*-factors(gt) *etc*. and is not relevant to the choice of reflections for refinement. *R*-factors based on *F*^2^ are statistically about twice as large as those based on *F*, and *R*- factors based on ALL data will be even larger.

### Fractional atomic coordinates and isotropic or equivalent isotropic displacement parameters (Å^2^)

**Table:**

	*x*	*y*	*z*	*U*_iso_*/*U*_eq_	
O11	0.88666 (17)	0.45353 (18)	0.30827 (9)	0.0367 (3)	
O22	0.58826 (18)	0.34392 (18)	0.40929 (9)	0.0388 (3)	
O1	0.95392 (19)	0.29323 (18)	0.55962 (10)	0.0375 (3)	
O12	1.12854 (18)	0.55986 (19)	0.25411 (10)	0.0399 (3)	
C14	1.0751 (2)	0.3590 (2)	0.15808 (11)	0.0271 (3)	
C17	1.0257 (2)	0.4686 (2)	0.24760 (11)	0.0271 (3)	
N13	0.9603 (2)	0.2639 (2)	0.15315 (11)	0.0384 (3)	
N11	1.1556 (3)	0.1439 (3)	0.00732 (13)	0.0535 (5)	
C15	1.2310 (3)	0.3528 (3)	0.08744 (12)	0.0363 (4)	
H15	1.3087	0.4217	0.0893	0.044*	
C12	1.0059 (3)	0.1634 (3)	0.07720 (16)	0.0536 (5)	
H12	0.9239	0.0998	0.0724	0.064*	
C16	1.2665 (3)	0.2393 (3)	0.01366 (14)	0.0458 (5)	
H16	1.3732	0.2302	−0.0334	0.055*	
Li1	0.7612 (4)	0.4527 (4)	0.4490 (2)	0.0339 (6)	
Li2	0.7150 (4)	0.3328 (4)	0.2642 (2)	0.0371 (6)	
N23	0.6007 (2)	0.10648 (19)	0.28871 (10)	0.0326 (3)	
C27	0.4559 (2)	0.27807 (19)	0.43070 (11)	0.0253 (3)	
C24	0.4582 (2)	0.14055 (19)	0.36333 (11)	0.0249 (3)	
C25	0.3226 (2)	0.0542 (2)	0.37941 (12)	0.0313 (3)	
H25	0.2217	0.0804	0.4303	0.038*	
C22	0.6095 (3)	−0.0203 (3)	0.23334 (14)	0.0415 (4)	
H22	0.7098	−0.0463	0.1822	0.050*	
O21	0.32633 (17)	0.31149 (16)	0.50106 (9)	0.0336 (3)	
N21	0.4884 (2)	−0.1138 (2)	0.24405 (12)	0.0405 (4)	
C26	0.3446 (3)	−0.0731 (2)	0.31605 (14)	0.0383 (4)	
H26	0.2546	−0.1326	0.3244	0.046*	
O2	0.5018 (2)	0.55144 (19)	0.18214 (11)	0.0402 (3)	
H1	0.922 (4)	0.340 (3)	0.614 (2)	0.055 (7)*	
H3	0.378 (4)	0.549 (4)	0.208 (2)	0.068 (8)*	
H2	1.071 (5)	0.274 (4)	0.531 (2)	0.080 (9)*	
H4	0.505 (5)	0.649 (5)	0.189 (3)	0.085 (10)*	

### Atomic displacement parameters (Å^2^)

**Table:**

	*U*^11^	*U*^22^	*U*^33^	*U*^12^	*U*^13^	*U*^23^
O11	0.0329 (6)	0.0583 (7)	0.0359 (6)	−0.0296 (5)	0.0146 (4)	−0.0274 (5)
O22	0.0413 (6)	0.0582 (8)	0.0401 (6)	−0.0374 (6)	0.0153 (5)	−0.0283 (5)
O1	0.0344 (6)	0.0499 (7)	0.0410 (6)	−0.0248 (5)	0.0082 (5)	−0.0217 (5)
O12	0.0398 (6)	0.0590 (7)	0.0429 (6)	−0.0357 (6)	0.0152 (5)	−0.0297 (6)
C14	0.0279 (7)	0.0358 (7)	0.0252 (6)	−0.0174 (6)	0.0055 (5)	−0.0138 (5)
C17	0.0252 (6)	0.0375 (7)	0.0259 (6)	−0.0167 (6)	0.0040 (5)	−0.0138 (5)
N13	0.0384 (7)	0.0538 (8)	0.0413 (7)	−0.0309 (7)	0.0145 (6)	−0.0278 (6)
N11	0.0659 (11)	0.0736 (11)	0.0482 (9)	−0.0461 (10)	0.0266 (8)	−0.0421 (8)
C15	0.0386 (8)	0.0528 (10)	0.0323 (7)	−0.0300 (7)	0.0132 (6)	−0.0202 (7)
C12	0.0612 (12)	0.0773 (13)	0.0555 (11)	−0.0511 (11)	0.0262 (9)	−0.0457 (10)
C16	0.0511 (10)	0.0667 (12)	0.0372 (8)	−0.0364 (10)	0.0230 (8)	−0.0293 (8)
Li1	0.0348 (13)	0.0468 (15)	0.0335 (13)	−0.0252 (12)	0.0086 (10)	−0.0205 (11)
Li2	0.0364 (14)	0.0545 (16)	0.0374 (13)	−0.0302 (13)	0.0131 (11)	−0.0254 (12)
N23	0.0345 (7)	0.0371 (7)	0.0376 (7)	−0.0215 (5)	0.0107 (5)	−0.0206 (5)
C27	0.0252 (6)	0.0305 (7)	0.0256 (6)	−0.0141 (5)	0.0016 (5)	−0.0107 (5)
C24	0.0258 (7)	0.0260 (6)	0.0278 (6)	−0.0132 (5)	0.0013 (5)	−0.0094 (5)
C25	0.0307 (7)	0.0335 (7)	0.0376 (8)	−0.0196 (6)	0.0069 (6)	−0.0119 (6)
C22	0.0487 (10)	0.0469 (9)	0.0451 (9)	−0.0298 (8)	0.0187 (8)	−0.0286 (8)
O21	0.0311 (6)	0.0429 (6)	0.0360 (6)	−0.0193 (5)	0.0110 (4)	−0.0213 (5)
N21	0.0517 (9)	0.0387 (8)	0.0460 (8)	−0.0280 (7)	0.0072 (7)	−0.0212 (6)
C26	0.0469 (9)	0.0379 (8)	0.0448 (9)	−0.0297 (7)	0.0034 (7)	−0.0142 (7)
O2	0.0412 (7)	0.0468 (7)	0.0485 (7)	−0.0290 (6)	0.0118 (5)	−0.0239 (5)

### Geometric parameters (Å, º)

**Table:**

O11—C17	1.2491 (18)	N11—C12	1.332 (2)
Li1—O11	1.961 (3)	C15—C16	1.386 (2)
Li1—O22	1.932 (3)	C15—H15	0.9300
Li1—O21^i^	1.953 (3)	C12—H12	0.9300
Li1—O1	1.967 (3)	C16—H16	0.9300
Li2—O11	2.021 (3)	N23—C22	1.3299 (19)
Li2—O22	2.020 (3)	N23—C24	1.3370 (18)
Li2—N13	2.155 (3)	C27—O21	1.2395 (17)
Li2—O2	1.998 (4)	C27—C24	1.5257 (18)
Li2—N23	2.205 (3)	C24—C25	1.3803 (19)
O22—C27	1.2463 (17)	C25—C26	1.383 (2)
O1—H1	0.83 (3)	C25—H25	0.9300
O1—H2	0.87 (3)	C22—N21	1.335 (2)
O12—C17	1.2425 (18)	C22—H22	0.9300
C14—N13	1.3354 (18)	O21—Li1^i^	1.953 (3)
C14—C15	1.381 (2)	N21—C26	1.323 (2)
C14—C17	1.5263 (19)	C26—H26	0.9300
N13—C12	1.332 (2)	O2—H3	0.93 (3)
N11—C16	1.318 (2)	O2—H4	0.81 (4)
			
C17—O11—Li1	149.71 (13)	O2—Li2—O22	98.68 (14)
C17—O11—Li2	117.79 (12)	O2—Li2—O11	102.08 (15)
Li1—O11—Li2	91.26 (11)	O22—Li2—O11	86.21 (11)
C27—O22—Li1	151.27 (13)	O2—Li2—N13	103.79 (15)
C27—O22—Li2	116.48 (12)	O22—Li2—N13	155.06 (18)
Li1—O22—Li2	92.15 (11)	O11—Li2—N13	78.69 (10)
Li1—O1—H1	108.4 (17)	O2—Li2—N23	104.14 (13)
Li1—O1—H2	106 (2)	O22—Li2—N23	77.76 (10)
H1—O1—H2	121 (3)	O11—Li2—N23	150.98 (18)
N13—C14—C15	121.20 (13)	N13—Li2—N23	106.61 (13)
N13—C14—C17	115.74 (12)	C22—N23—C24	116.37 (13)
C15—C14—C17	123.04 (13)	C22—N23—Li2	134.44 (13)
O12—C17—O11	126.77 (13)	C24—N23—Li2	107.11 (11)
O12—C17—C14	117.50 (12)	O21—C27—O22	126.68 (13)
O11—C17—C14	115.71 (13)	O21—C27—C24	117.70 (12)
C12—N13—C14	116.22 (14)	O22—C27—C24	115.61 (12)
C12—N13—Li2	133.18 (14)	N23—C24—C25	121.87 (13)
C14—N13—Li2	110.24 (12)	N23—C24—C27	116.20 (12)
C16—N11—C12	115.34 (15)	C25—C24—C27	121.93 (13)
C14—C15—C16	117.12 (15)	C24—C25—C26	116.60 (14)
C14—C15—H15	121.4	C24—C25—H25	121.7
C16—C15—H15	121.4	C26—C25—H25	121.7
N11—C12—N13	127.28 (16)	N23—C22—N21	126.36 (15)
N11—C12—H12	116.4	N23—C22—H22	116.8
N13—C12—H12	116.4	N21—C22—H22	116.8
N11—C16—C15	122.80 (15)	C27—O21—Li1^i^	120.24 (12)
N11—C16—H16	118.6	C26—N21—C22	116.08 (13)
C15—C16—H16	118.6	N21—C26—C25	122.67 (14)
O22—Li1—O21^i^	125.60 (16)	N21—C26—H26	118.7
O22—Li1—O11	90.34 (12)	C25—C26—H26	118.7
O21^i^—Li1—O11	115.37 (15)	Li2—O2—H3	108.4 (17)
O22—Li1—O1	115.40 (15)	Li2—O2—H4	112 (2)
O21^i^—Li1—O1	99.05 (12)	H3—O2—H4	108 (3)
O11—Li1—O1	111.64 (15)		

Symmetry code: (i) −*x*+1, −*y*+1, −*z*+1.

### Hydrogen-bond geometry (Å, º)

**Table:**

*D*—H···*A*	*D*—H	H···*A*	*D*···*A*	*D*—H···*A*
O1—H1···O12^ii^	0.83 (3)	1.98 (3)	2.8120 (18)	175 (2)
O2—H3···O12^iii^	0.93 (3)	1.84 (3)	2.7671 (18)	177 (3)
O1—H2···O21^iv^	0.87 (3)	1.98 (3)	2.7941 (18)	155 (3)
O2—H4···N21^v^	0.81 (4)	2.10 (4)	2.8881 (19)	166 (3)

Symmetry codes: (ii) −*x*+2, −*y*+1, −*z*+1; (iii) *x*−1, *y*, *z*; (iv) *x*+1, *y*, *z*; (v) *x*, *y*+1, *z*.
